# Supplementary material for: Neural prediction decorrelation reveals that adversarial robustness substantially improves DNN prediction accuracy across the entire human auditory cortex
Source: bioRxiv. 2026 Oct 11:2026.08.05.743059. Preprint. [Version 2] doi: 10.64898/2026.08.05.743059 (PMC13691987; doi:10.64898/2026.08.05.743059)
Supplement: Supplementary file 1 [file NIHPP2026.08.05.743059V2-supplement-1.pdf]

bioRxiv preprint doi: https://doi.org/10.64898/2026.08.05.743059; this version posted October 11, 2026. The copyright holder for this preprint (which was not certified by peer review) is the author/funder, who has granted bioRxiv a license to display the preprint in perpetuity. It is made available under a CC-BY 4.0 International license.

Supplement

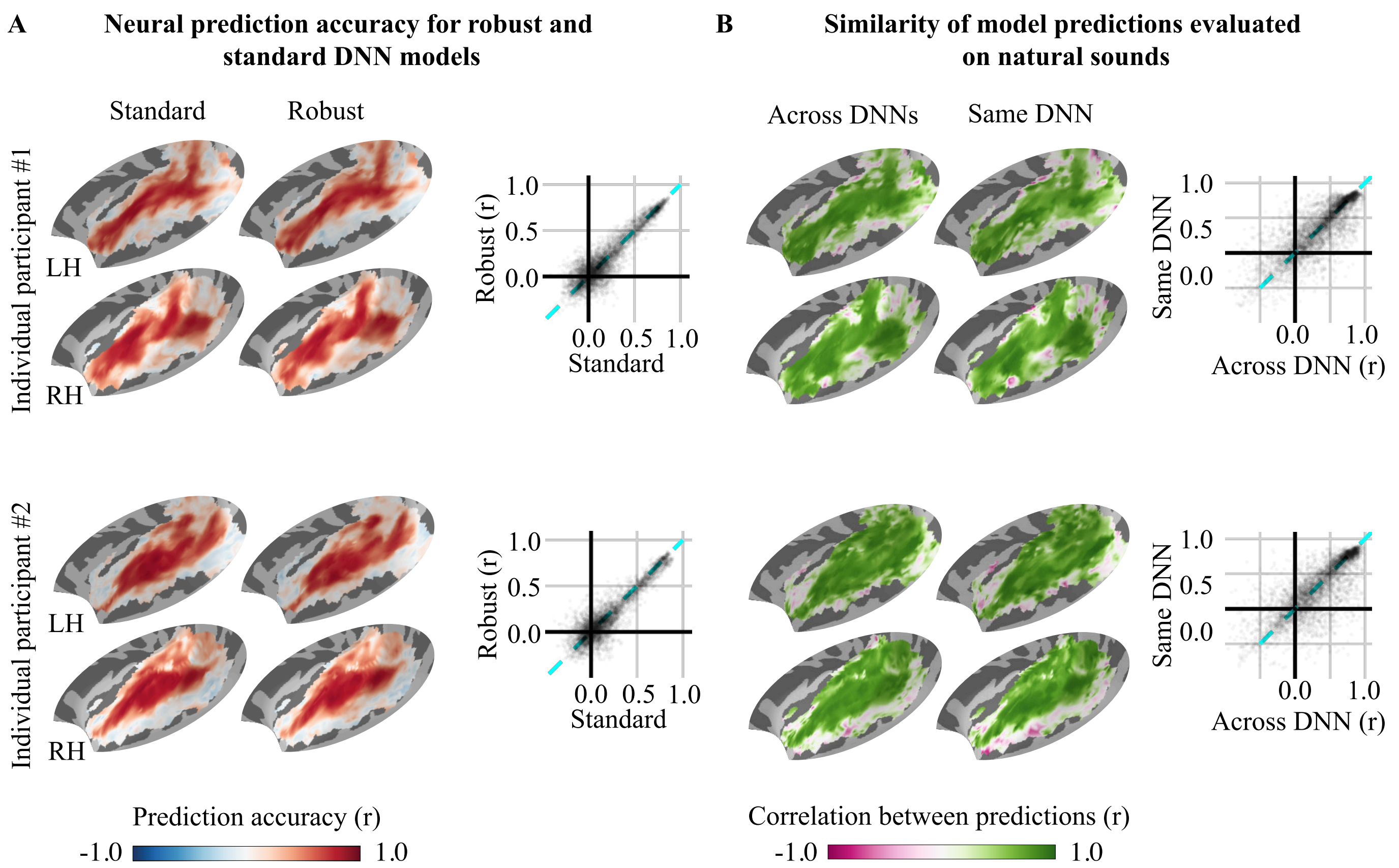

Fig. S1: **A** Prediction accuracy (Pearson correlation) for standard (left) and adversarially robust (right) models for individual participants. The scatter plot compares the prediction accuracies of the standard (x-axis) and adversarially robust (y-axis) models for all voxels analyzed. **B** Similarity of the adversarially robust and standard model predictions obtained by correlating the model predictions across models for each voxel ("Across DNNs", left). The models shown here use different DNNs and were fit to neural responses using non-overlapping training stimuli. This allows us to plot a measure of the maximum possible correlation computed by correlating the predictions from two models with the same DNN (e.g., standard vs. standard, robust vs. robust) fit to neural responses using non-overlapping stimulus sets ("Same DNN", right). The scatter plot compares the Across DNN correlations (x-axis) with the Same DNN correlations (y-axis) for all analyzed voxels.

bioRxiv preprint doi: https://doi.org/10.64898/2026.08.05.743059; this version posted October 11, 2026. The copyright holder for this preprint (which was not certified by peer review) is the author/funder, who has granted bioRxiv a license to display the preprint in perpetuity. It is made available under a CC-BY 4.0 International license.

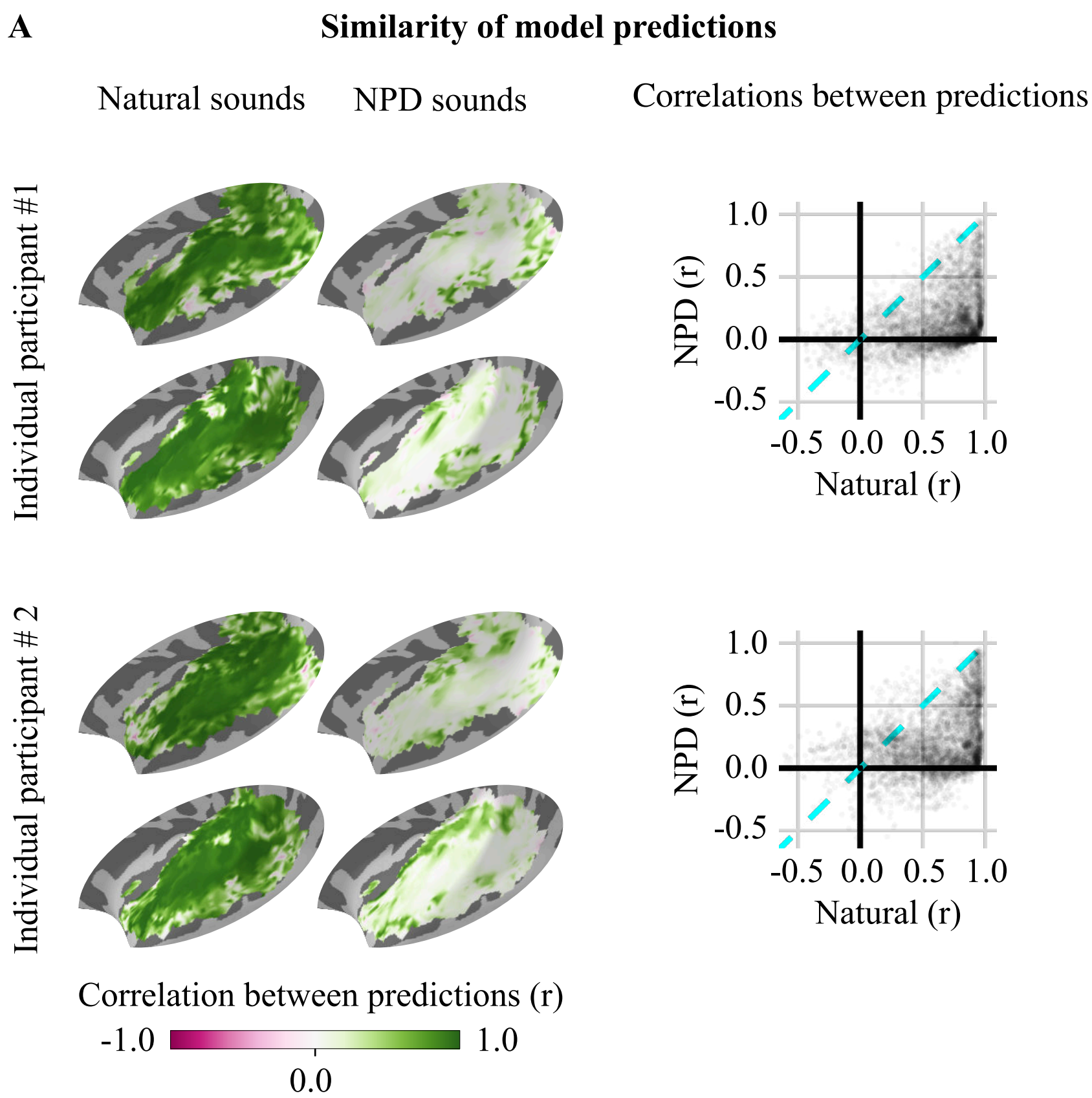

Fig. S2: **A** Similarity of model predictions for individual participants. Correlations between model predictions are shown for natural (left map) and NPD (right map) stimuli. The scatter plot compares correlations between model predictions for the natural sounds (x-axis) and NPD (y-axis) sounds.

bioRxiv preprint doi: https://doi.org/10.64898/2026.08.05.743059; this version posted October 11, 2026. The copyright holder for this preprint (which was not certified by peer review) is the author/funder, who has granted bioRxiv a license to display the preprint in perpetuity. It is made available under a CC-BY 4.0 International license.

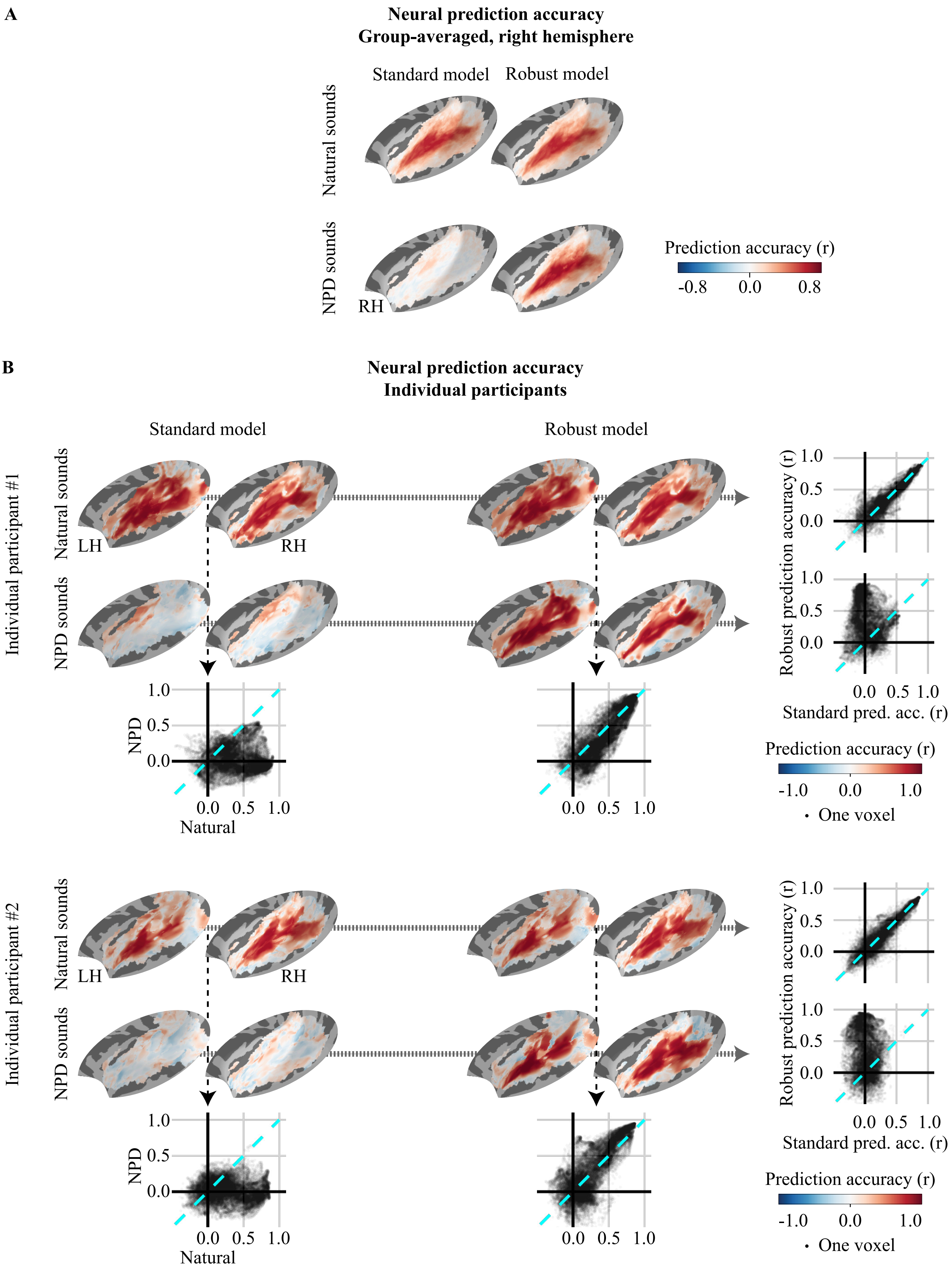

Fig. S3: **A** Group-averaged neural prediction accuracy for natural and NPD sounds in the right hemisphere. Prediction accuracy is shown for the standard (left column) and robust (right column) models for natural (top row) and NPD (bottom row) sounds. **B** Prediction accuracy for natural and NPD sounds for two individual participants. Prediction accuracy of the standard (left column) and adversarially robust (right column) models for natural (top rows) and NPD (bottom rows) sounds. The bottom scatter plots compare the prediction accuracy for natural and NPD sounds within a model (e.g., standard vs. standard). The scatter plots on the right compare across models (e.g., adversarially robust vs. standard predictions of natural (top) and NPD (bottom) sounds).

bioRxiv preprint doi: https://doi.org/10.64898/2026.08.05.743059; this version posted October 11, 2026. The copyright holder for this preprint (which was not certified by peer review) is the author/funder, who has granted bioRxiv a license to display the preprint in perpetuity. It is made available under a CC-BY 4.0 International license.

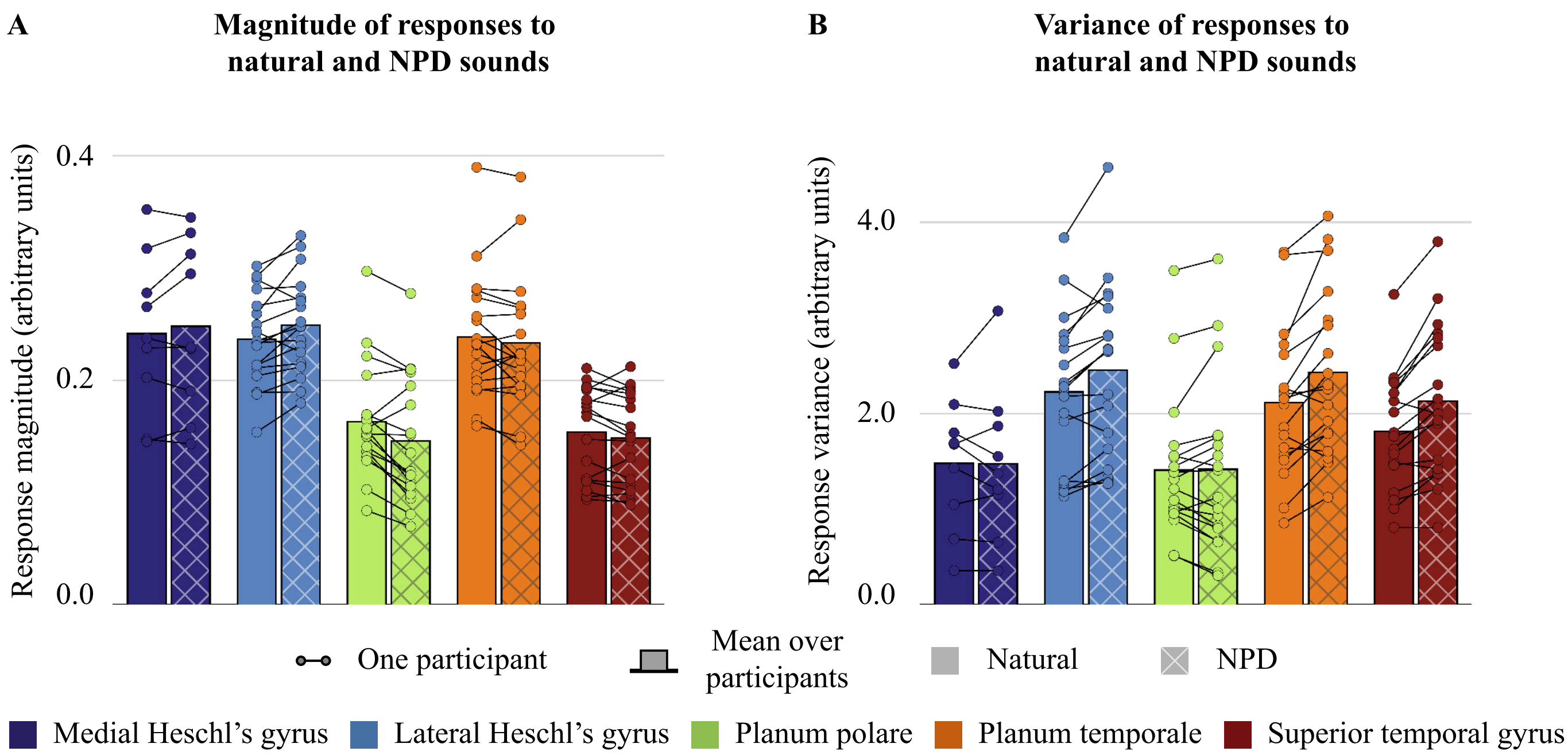

Fig. S4: **A** Magnitude of measured responses to natural and NPD sounds in each ROI. Circles show the median response across voxels for each ROI for a single participant. Bar plots show the mean across participants for natural sounds (solid) and NPD sounds (cross-hatched). **B** Variance of measured responses to natural and NPD sounds in each ROI. Same format as panel A.

CochResNet50 Architecture:

Model Stage	PyTorch Operation	Candidate during layer selection?	Output Shape
natural_audio	Input (waveform)	No	(40000)
cochleagram	Cochleagram<br>Conv2d(1, 64, kernel_size=7, stride=2, padding=3, bias=False)<br>BatchNorm2d(64)	No<br>No<br>No	(1,211,390)<br>(64, 106, 195)<br>(64, 106, 195)
conv1_relu1	ReLU<br>MaxPool2d(kernel_size=3, stride=2, padding=1)	No<br>No	(64, 106, 195)<br>(64, 53, 98)
layer1	ResNetBlock(inplanes=64, planes=64, num_blocks=3, stride=1)	Yes	(256, 53, 98)
layer2	ResNetBlock(inplanes=256, planes=128, num_blocks=4, stride=2)	Yes	(512, 27, 49)
layer3	ResNetBlock(inplanes=512, planes=256, num_blocks=6, stride=2)	Yes	(1024, 14, 25)
layer4	ResNetBlock(inplanes=1024, planes=512, num_blocks=3, stride=2)	Yes	(2048, 7, 13)
avgpool	AdaptiveAvgPool2d(1,1)	Yes	(2048, 1, 1)
final	Linear(2048, 794)	No	(794)

ResNetBlock component architecture:

1.	input (x)
2.	1×1 Conv2d(inplanes, planes, stride=1)
3.	BatchNorm2d(planes)
4.	ReLU
5.	3×3 Conv2d (planes, planes, stride=1)
6.	BatchNorm2d(planes)
7.	ReLU
8.	1×1 Conv2d (planes, planes $\times$ expansion, stride=1)
9.	BatchNorm2d(planes)
10.	Residual connection on x (if inplanes $\neq$ planes $\times$ expansion): 1×1 Conv2D (inplanes, planes $\times$ expansion, stride)
11.	Residual connection on x (if inplanes $\neq$ planes $\times$ expansion): BatchNorm2d(planes $\times$ expansion)
12.	Add output from (9) to output from (11)
13.	(Output) ReLU

Table ST1: CochResnet50 architecture. The DNN takes a 2-dimensional (time $\times$ frequency) cochleagram representation of sound as input and processes it using a ResNet50 backbone architecture (He et al. 2016). Each ResNetBlock consists of num_blocks copies of the ResNetBlock component architecture above, stacked together to form a single ResNetBlock. The expansion factor was set to four for all layers. Only the outputs of layer1, layer2, layer3, and layer4 were considered during layer selection as part of the encoding model fitting (as indicated in the "Candidate during layer selection?" column; see Methods for details).

bioRxiv preprint doi: https://doi.org/10.64898/2026.08.05.743059; this version posted October 11, 2026. The copyright holder for this preprint (which was not certified by peer review) is the author/funder, who has granted bioRxiv a license to display the preprint in perpetuity. It is made available under a CC-BY 4.0 International license.

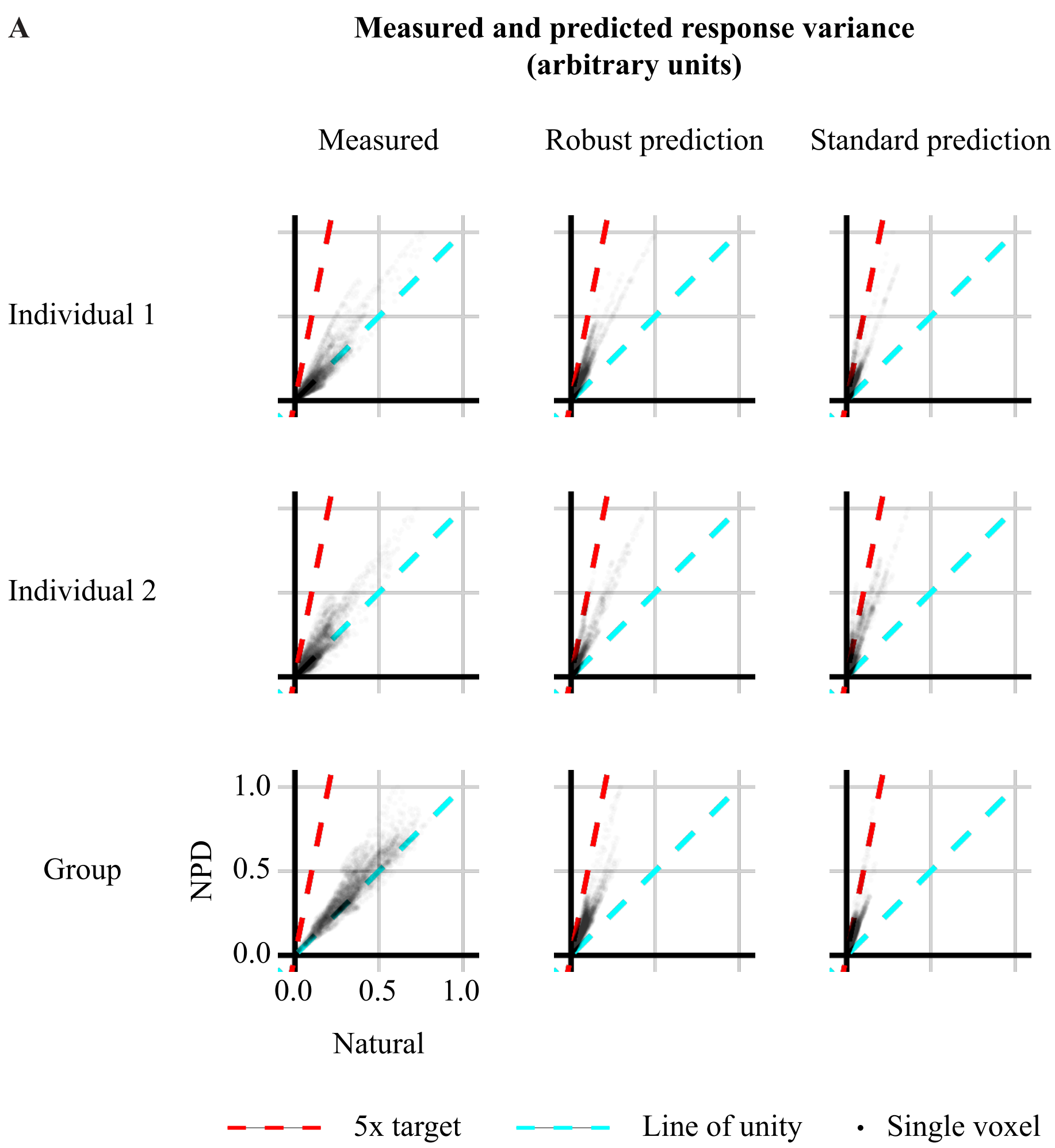

Fig. S5: **A** Voxel-wise variances of measured (left column) and predicted (middle and right columns) responses, for two individual participants (top and middle rows) and at the group level (bottom row). The 5× variance target is shown in each plot. We observe that NPD sounds tend to produce higher variance responses than natural sounds, as desired. This difference is smaller than the difference in predicted variance, plausibly because measured neural responses include noise that is independent of the stimuli.

bioRxiv preprint doi: https://doi.org/10.64898/2026.08.05.743059; this version posted October 11, 2026. The copyright holder for this preprint (which was not certified by peer review) is the author/funder, who has granted bioRxiv a license to display the preprint in perpetuity. It is made available under a CC-BY 4.0 International license.

A

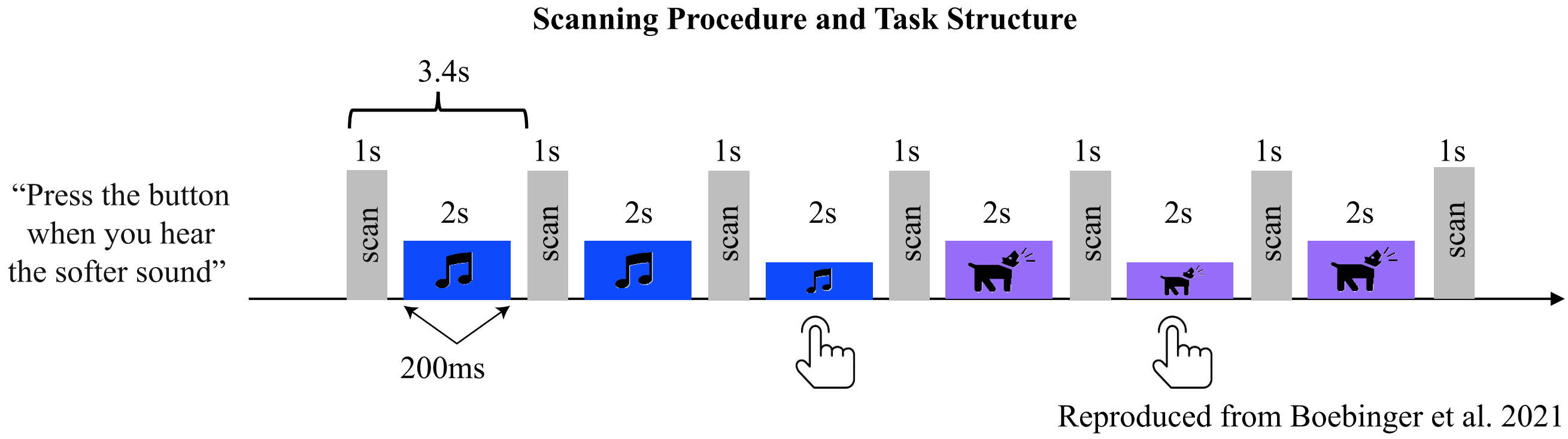

B

Scanning parameters

**Parameter**	**Value**
TR (ms)	3400
TA (ms)	1000
Delay in TR (ms)	1200
Echo time (ms)	30.0
Number of slices	52
In-plane voxel (mm)	2.6 x 2.6
Slice thickness (mm)	2.6
Gap between slices	0 %
MB acceleration	4
iPAT acceleration	1
Number of TRs per run	108
Number of runs	6
Head coil	64-channel

Fig. S6: **A** Schematic of the scanning procedure. Sounds were presented between scans to mitigate the effects of scanner noise. Scans had an acquisition time (TA) of 1 second, and the 2-second sounds were padded by 200 milliseconds of silence at onset and offset, resulting in a total repetition time (TR) of 3.4 seconds. Each sound was played three times in succession, and either the second or third presentation was at a lower volume. Participants were tasked with pressing a button upon recognizing the quieter presentation. **B** fMRI scan parameters.
